# Explainable AI for Intraoperative Motor-Evoked Potential Muscle Classification in Neurosurgery: Bicentric Retrospective Study

**DOI:** 10.2196/63937

**Published:** 2025-03-24

**Authors:** Qendresa Parduzi, Jonathan Wermelinger, Simon Domingo Koller, Murat Sariyar, Ulf Schneider, Andreas Raabe, Kathleen Seidel

**Affiliations:** 1 Graduate School for Health Sciences University of Bern Bern Switzerland; 2 Department of Neurosurgery Inselspital Bern University Hospital, University of Bern Bern Switzerland; 3 Department of Neurosurgery Lucerne Cantonal Hospital Lucerne Switzerland; 4 School of Engineering and Computer Science Bern University of Applied Sciences Biel Switzerland

**Keywords:** intraoperative neuromonitoring, motor evoked potential, artificial intelligence, machine learning, deep learning, random forest, convolutional neural network, explainability, medical informatics, personalized medicine, neurophysiological, monitoring, orthopedic, motor, neurosurgery

## Abstract

**Background:**

Intraoperative neurophysiological monitoring (IONM) guides the surgeon in ensuring motor pathway integrity during high-risk neurosurgical and orthopedic procedures. Although motor-evoked potentials (MEPs) are valuable for predicting motor outcomes, the key features of predictive signals are not well understood, and standardized warning criteria are lacking. Developing a muscle identification prediction model could increase patient safety while allowing the exploration of relevant features for the task.

**Objective:**

The aim of this study is to expand the development of machine learning (ML) methods for muscle classification and evaluate them in a bicentric setup. Further, we aim to identify key features of MEP signals that contribute to accurate muscle classification using explainable artificial intelligence (XAI) techniques.

**Methods:**

This study used ML and deep learning models, specifically random forest (RF) classifiers and convolutional neural networks (CNNs), to classify MEP signals from routine supratentorial neurosurgical procedures from two medical centers according to muscle identity of four muscles (extensor digitorum, abductor pollicis brevis, tibialis anterior, and abductor hallucis). The algorithms were trained and validated on a total of 36,992 MEPs from 151 surgeries in one center, and they were tested on 24,298 MEPs from 58 surgeries from the other center. Depending on the algorithm, time-series, feature-engineered, and time-frequency representations of the MEP data were used. XAI techniques, specifically Shapley Additive Explanation (SHAP) values and gradient class activation maps (Grad-CAM), were implemented to identify important signal features.

**Results:**

High classification accuracy was achieved with the RF classifier, reaching 87.9% accuracy on the validation set and 80% accuracy on the test set. The 1D- and 2D-CNNs demonstrated comparably strong performance. Our XAI findings indicate that frequency components and peak latencies are crucial for accurate MEP classification, providing insights that could inform intraoperative warning criteria.

**Conclusions:**

This study demonstrates the effectiveness of ML techniques and the importance of XAI in enhancing trust in and reliability of artificial intelligence–driven IONM applications. Further, it may help to identify new intrinsic features of MEP signals so far overlooked in conventional warning criteria. By reducing the risk of muscle mislabeling and by providing the basis for possible new warning criteria, this study may help to increase patient safety during surgical procedures.

## Introduction

The importance of intraoperative neurophysiological monitoring (IONM) during high-risk neurosurgical and orthopedic procedures has been established in recent decades [1]. In particular, the monitoring of motor evoked potentials (MEPs) helps to assess the functional integrity of motor pathways during surgeries and allows postoperative motor outcomes to be predicted [2-9]. However, the features of this complex signal that contribute to its predictive potential are still poorly understood and there are few standardized warning criteria to alert the surgeon. Currently, the best-established and most reliable MEP warning criterion during IONM is a 50% drop in amplitude [10].

Because changes in MEP amplitude are predictive of postoperative motor outcome, it is natural to ask whether other properties of the signals could be important for decision-making. The emergence of machine learning (ML) methods has led to an interest in leveraging these techniques to classify MEPs in the hope of improving intraoperative decision-making [11,12]. However, most of the studies so far have focused on identifying the most accurate and robust ML algorithms rather than on uncovering the underlying patterns leading to the decisions.

In our previous work, we established prediction algorithms for muscle identification to provide a proof of principle within a solid ground truth framework before translating them to outcome predictions [12]. Meanwhile, Boaro et al [13] implemented a similar classification task with additional ML models and muscles. The robustness of ML algorithms on clinical data needs to be established using independent data sources, which is why we have expanded our data set to include signals from an external validation center.

To gain a deeper understanding of our signals, we investigated them in both the time-series and time-frequency domains, which have been shown to be useful in the quantification of disease-related MEP changes [14]. In addition to the standard ML models, we used deep learning methods to leverage their power of internal feature representation. Although these algorithms can accurately predict the identity of muscles based on MEP signals, the specific criteria that these algorithms use to make their predictions are not well understood [15-17]. For research in the field of IONM, this explanatory information is probably at least as important as the predictions themselves, as it can provide new insights into the mechanisms of neurophysiological changes. For this reason, we used methods from the emerging field of explainable artificial intelligence (XAI) [18,19]. The aim was to combine methods to ensure comprehensive interpretability of our different models’ decisions.

Our study provides a robust framework for classifying MEPs recorded in routine neurosurgical procedures according to their muscle identity with high accuracy and we validated the methods using data from two independent study centers. Importantly, we elucidate the decision-making processes of our ML models through post hoc analyses, thereby enabling their effective application to previously unseen data and novel situations. These algorithms could act as a safety mechanism in the operating room by detecting mislabeling of muscles and by focusing on new intrinsic features of MEPs. Thus, they may enhance the usage and acceptance of artificial intelligence (AI) in medical decision-making through their interpretability.

## Methods

### Ethical Considerations

This retrospective study was approved by the cantonal ethics committee of Bern, Switzerland (BASEC-ID 2023–00277). All included patients gave their informed written consent for further use and publication of their anonymized data. The routinely collected data are dated from 2018 to 2022 and coded for the analysis of this paper. Only patients over the age of 18 were included in the study, all received neurosurgical interventions and were not stratified according to their clinical outcome since the outcome prediction of the study was muscle identification. The muscle recordings are set routinely independent of sociodemographic or clinical factors.

In the following, we describe the datasets collected, the preselection approach applied to the data, the methods used for signal data representation, and the analytical techniques used, including XAI approaches to elucidate MEP feature importance.

### MEP Data and Signal Recordings

**Figure 1:**
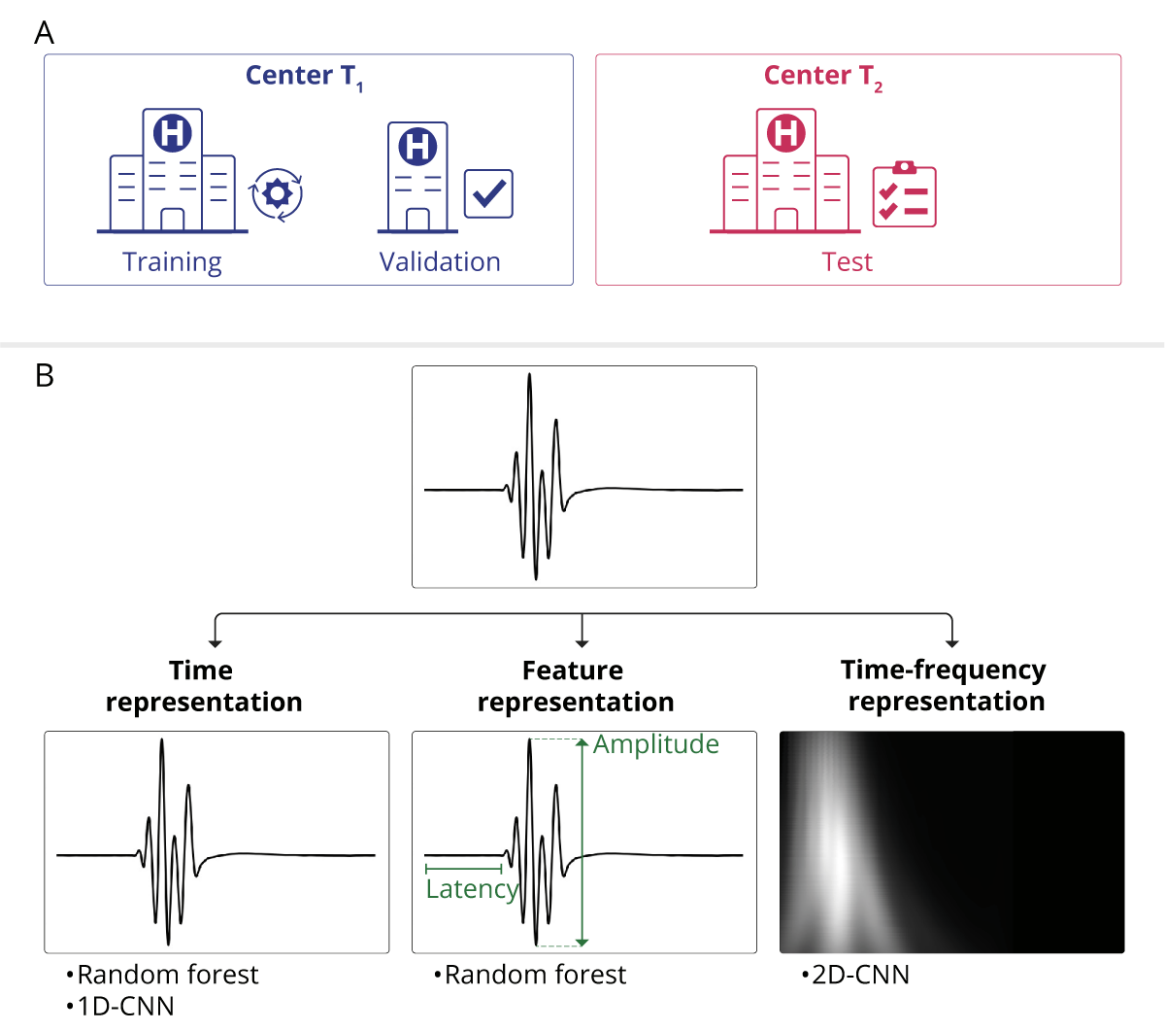
Data analysis pipeline. (A) Bicentric training, validation, and testing setup. (B) Data representation and the algorithms used on each representation. CNN: convolutional neural network.

The MEP data used in this study were obtained during routine neurosurgical procedures and were retrospectively collected and analyzed. This study was exploratory in nature, and no formal protocol was prepared. Recordings from 151 surgeries on 144 patients at one center (Inselspital, University Hospital Bern, Switzerland), “center T_1_” were used for training and validation, and recordings from 58 surgeries on 57 patients at an independent center (Cantonal Hospital in Lucerne, Switzerland), “center T_2_” were used for testing (see Figure 1A). In total, there were 94 females and 107 males and the median age at surgery was 61 years (see Table 1 for distribution between centers). Overall, 182 patients underwent surgery for a brain tumor and 19 for vascular pathologies. The total number of MEP signals was 36,992 for center T_1_ and 24,298 for center T_2_, with at least 3000 samples for each predicted class (see Table 1 for detailed information on classes). This sample size was determined by the number of routine interventions and ensures that any complexity of the ML models used can appropriately be trained, tested, and validated.

IONM was performed according to a standardized protocol, as previously described [8,20]. The MEPs were elicited either through transcranial electric stimulation (TES) via corkscrew electrodes or direct cortical stimulation (DCS) via strip electrodes placed directly on the cortex and recorded via needle electrodes in the muscle belly. Stimulation to elicit MEPs was conducted under general anesthesia using a train of 5 anodal stimuli with a pulse duration of 0.5 milliseconds, and an interstimulus interval of 4 milliseconds, known as the short train method. Both centers used ISIS Systems to record the MEPs. The sampling frequency was set at 20 kHz, with hardware high- and low-pass filters at 30 Hz and 5 kHz, respectively. TES was used in all the surgeries performed in center T_1_ and in 95% (55 of 58) of those performed in center T_2_ to elicit MEPs. In addition, DCS was used to elicit MEPs in 134 of the 151 (89%) surgeries performed in center T_1_ and 17 of 58 (30%) surgeries performed in center T_2_. The recordings consist of 2000 data points, corresponding to 100-millisecond windows for each signal. We selected MEP signals from the following 4 muscles: Extensor digitorum (EXT), abductor pollicis brevis (APB), tibialis anterior (TA), and abductor hallucis (AH). These muscles are routinely monitored during supratentorial surgeries, and the corresponding signals were available for most of the included patients from both sides. As the neurophysiologist labels the recording channels at the start of the surgery, the MEP data are automatically labeled upon saving.

We used custom-made Python 3.0 scripts for all the data analysis and classification tasks.

**Table 1:** Surgery, patient, and recording characteristics. The percentage of clinical outcome is calculated in relation to the total number of patients, while the percentage of TES and DCS stimulations is calculated with respect to the total number of surgeries (including redo operations).

Categories				Center T_1_		Center T_2_
**Demography, n**						
		Patients	144		57	
		M^a^	78		29	
		F^b^	66		28	
		Age^c^	62		58	
**Pathology, n**						
	Meningioma		0		8	
	Schwannoma		0		2	
	Oligodendroglioma		7		2	
	Astrocytoma		16		10	
	Glioblastoma		73		10	
	Metastasis		44		4	
	Aneurysm		0		13	
	AVM^d^		1		4	
	Cavernoma		2		1	
	Trigeminal neuralgia		0		3	
	Radio necrosis		1		0	
**Clinical outcome, n (%)**						
	Deficits at discharge		29 (19)		9 (16)	
	Deficits at follow-up		9 (6)		4 (7)	
**Neurophysiology**						
	Surgeries, n		151		58	
	TES^e^ stimulation, n (%)		151 (100)		55 (95)	
	DCS^f^ stimulation, n (%)		134 (89)		17 (30)	
	MEP^g^ signals, n		36,992		24,298	
	EXT^h^ signals, n (%)		11,958 (31.8)		3628 (14.5)	
	APB^i^ signals, n (%)		15,800 (42.9)		10,670 (42.6)	
	TA^j^ signals, n (%)		5773 (15.7)		4970 (21.4)	
	AH^k^ signals, n (%)		3461 (9.6)		5030 (21.6)	

^a^M: male.

^b^F: female.

^c^Age is the median age of all patients.

^d^AVM: arteriovenous malformation.

^e^TES: transcranial electric stimulation.

^f^DCS: direct cortical stimulation.

^g^MEP: motor-evoked potential.

^h^EXT: extensor digitorum.

^i^APB: abductor pollicis brevis.

^j^TA: tibialis anterior.

^k^AH: abductor hallucis.

### Preprocessing and Data Representation

#### Preselection

An automatic MEP selection algorithm was written to determine whether a given recording contained an MEP [12]. To remove stimulation artifacts from the train of 5, we excluded the first 400 data points (corresponding to 20 milliseconds). Then, two features were computed: the onset latency and duration of the signal. Onset latency is defined as 1 millisecond (empirically determined) before the trace crosses the mean of the baseline (the last 5 milliseconds of the recording) plus or minus the SD of the entire recording. The end of the signal was calculated similarly, by starting from the end of the recording. We defined the duration as the end of signal latency minus onset latency. In addition, the time interval between the first and last peak was determined using the scipy.signal function find_peaks. A recording was considered to contain an MEP if at least one peak was detected, the duration was less than 40 milliseconds, and the interval from the first to last peak was less than 35 milliseconds (in accordance with clinical experience).

In our analysis pipeline, we used three distinct representations of MEP data (see Figure 1B): time, feature, and time-frequency, each tailored to optimize the performance of our ML classifiers.

#### Time Representation

A finite-impulse response bandpass filter with 30- and 1000-Hz cutoff frequencies was applied to the 1600-dimensional signal vector. These frequency settings align with MEP visualization practices using the monitoring machine at center T_1_ (ie, the software filters). The signal vectors are then normalized with respect to the absolute maximum MEP value in each patient. This filtered and normalized time representation of the data was used to train, validate, and test a random forest (RF) classifier, as well as a 1-dimensional convolutional neural network (1D-CNN).

#### Feature Representation

We used a customized feature extraction algorithm to condense each filtered and normalized MEP signal into characteristic features. The initial choice of predictors is a combination of clinically used predictors (eg, latency, amplitude, minimum, and maximum) and routinely used features in general neurophysiological literature (spectral entropy, frequency, etc). After a correlation analysis (see Multimedia Appendix 1), we chose five features that showed no correlation describing relevant domains of the signal: peak latency, maximum signal value, number of peaks, main frequency, and slope.

Peak values were extracted with the scipy find_peak*s* function with peak prominence defined as twice the SD of the signal. The main frequency was calculated as the frequency at which the Fourier transform of the filtered data attained its maximum absolute value. Finally, the slope of the signal was computed as the mean of the first derivative of the signal (using the NumPy function gradient). The resulting 5-dimensional feature representation of the data was then used to train, validate, and test a RF classifier.

#### Time-Frequency Representation

Finally, employing the Python library PyWavelets (pywt), a continuous wavelet transform with a Mexican hat mother wavelet was applied to the data to transform them into 2D time-frequency representations. Scales ranging from 2 to 30 were logarithmically spaced, while the time dimension was undersampled to yield an array of dimensions 224×224. These 2D time-frequency representations were then used to train, validate, and test a 2D-CNN.

#### Statistical Testing

Using custom Python scripts, Student *t* tests were applied to compare the mean values of two different features. Specifically, for each muscle, the means of the different features were compared across the two centers. Statistical significance was set at *P*=.05.

#### Machine and Deep Learning Pipeline

The Python library scikit-learn [21] was used for the RF classifier, while tensorflow [22] and keras [23] were used to obtain the 1D- and 2D-CNN models. Hyperparameter tuning was carried out for the RF and 1D-CNN, while we used fixed parameters for the 2D-CNN (see below). The hyperparameters used in the grid search of the RF are the same as described in Multimedia Appendix 1. The architecture of our 1D-CNN is inspired by the model of Ahmed et al [24], and consists of two consecutive 1D convolutional layers, followed by a MaxPooling layer, a dropout layer, and a BatchNormalization layer. The model then gets flattened, before adding a dense layer and another dropout layer and finally ending in a dense output layer. The structure of our 2D-CNN is inspired by the model of Wang et al [25]. It consists of 4 blocks of 2D convolutional layers followed by BatchNormalization layers, with a MaxPooling layer after the second and third blocks and a GlobalMaxPooling layer after the fourth block. The model is capped off by a dense output layer. The specifics of these models are shown in Multimedia Appendix 1.

The dataset from Center T_1_ was split into 70% for training and 30% for validation (stratified according to patients), while the whole of the dataset from Center T_2_ was used for testing. In all cases, we used class weighting [26] to deal with the class imbalance problem (ie, the number of leg muscle MEPs is lower than the number of arm muscle MEPs, see Wermelinger et al [12] for a discussion of this issue).

#### Model Output and Outcome

**Figure 2:**
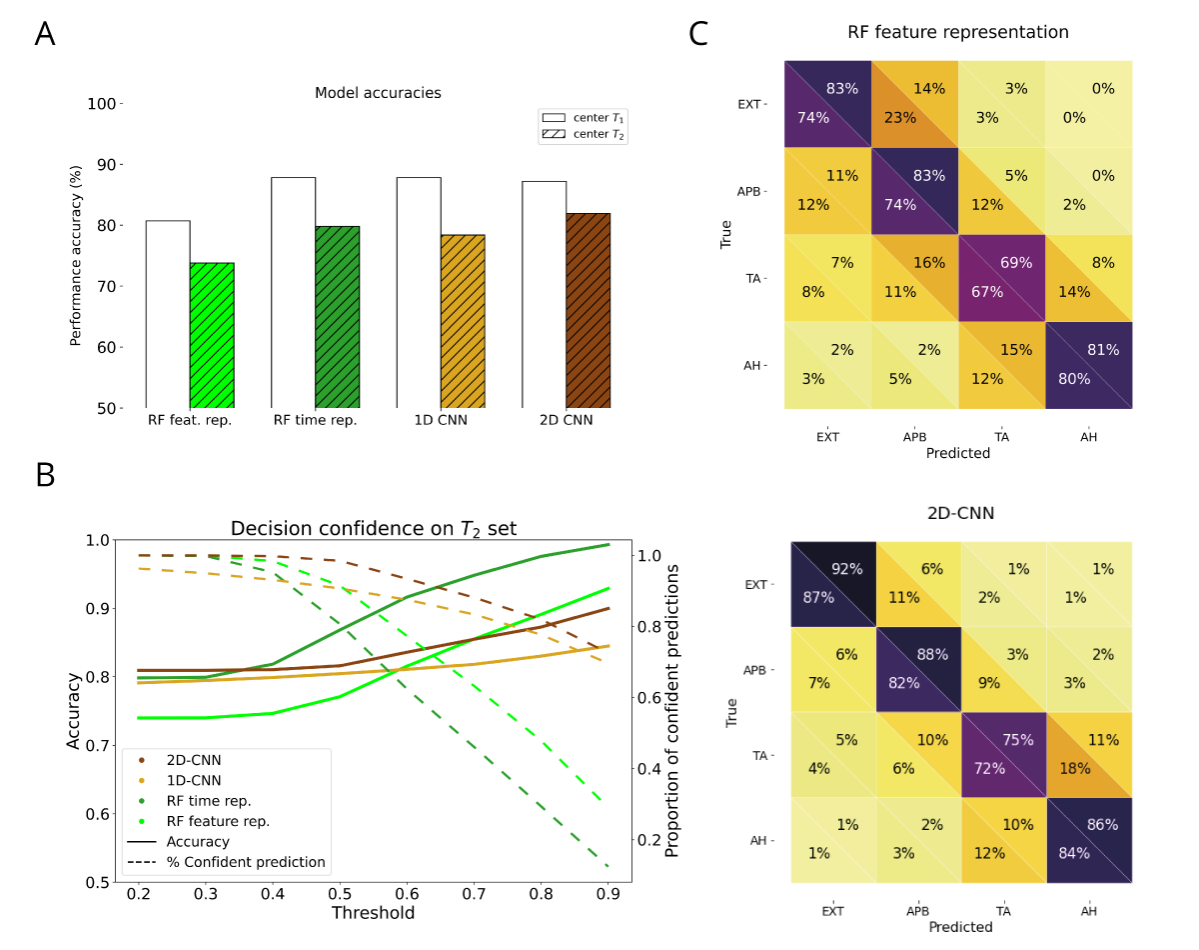
Classification results and confidence. (A) Validation accuracy (center T1, white) and test accuracy (center T2, colored) of all models. (B) Decision confidence. Solid lines are the accuracies (left y-axis) of the various models for different confidence thresholds. The dashed lines show the proportion of data with these confidences (right y-axis). RFs have a higher increase in accuracy compared with CNNs but at a higher data cost. (C) Bicentric confusion matrices: lower triangle (center T2), upper triangle (center T1) for both RF on feature representation (top) and 2D-CNN (bottom). The RF is slightly more congruent across centers than the 2D-CNN. CNN: convolutional neural network; RF: random forest.

All prediction models output probabilities of belonging to the predicted class. The decision thresholds were set at a chance level of 0.25 (likelihood of belonging to 1 of 4 classes of muscles). They were systematically explored and reported (see Figure 2B) for decision thresholds up to 0.9.

Accuracy was used as the primary performance metric, and the confusion matrix was used to evaluate the performance of the classification algorithm. The outcome assessment does not require subjective interpretation, since the muscle identity is objectively assessable, independent of sociodemographic background and clinical outcome.

No model updating or recalibration was performed during the model evaluation. While some variability in model performance was observed across different centers (Figure 2), we opted to retain the original model without adjustments. Future work may explore model updating to enhance performance in these areas.

#### Explainability

To elucidate how the RF classifiers made their decisions, we used feature importance and Shapley Additive Explanation (SHAP) values. Feature importance values are provided by the feature_importances*_* attribute of the scikit-learn RandomForestClassifier class, while the SHAP values are calculated with the SHAP library [27]. The feature importance values are determined by aggregating (mean and SD) the impurity decrease within each decision tree. SHAP values quantify the impact of each feature on prediction outcomes. Positive values signify a positive influence, while negative values indicate the opposite, with magnitude representing the strength of the effect. SHAP values of the RF on feature representation of the MEP data were computed on a random sample containing 20% (5919 MEPs) of the training data set. In the case of CNNs, we used gradient-weighted class activation mapping (Grad-CAM). This is a type of attention map, a visualization tool highlighting regions within an image considered by the neural network to be pivotal for specific predictions [28]. We adapted preexisting code to generate Grad-CAMs for both 1D- and 2D-CNNs [29,30]. The Grad-CAMs of all signals in the training data set were averaged to obtain the corresponding plots for the 1D-CNN (overall) and 2D-CNN (for each muscle).

## Results

### Differences and Similarities of MEP Properties Between Centers

Data from 151 surgeries from the training and validation center T_1_ yielded a total of 36,992 MEPs (11,958 EXT, 15,800 APB, 5773 TA, and 3461 AH), and the data from 58 surgeries from the test center T_2_ yielded a total of 24,298 MEPs (3628 EXT, 10,670 APB, 4970 TA, and 5030 AH; Table 1).

**Figure 3:**
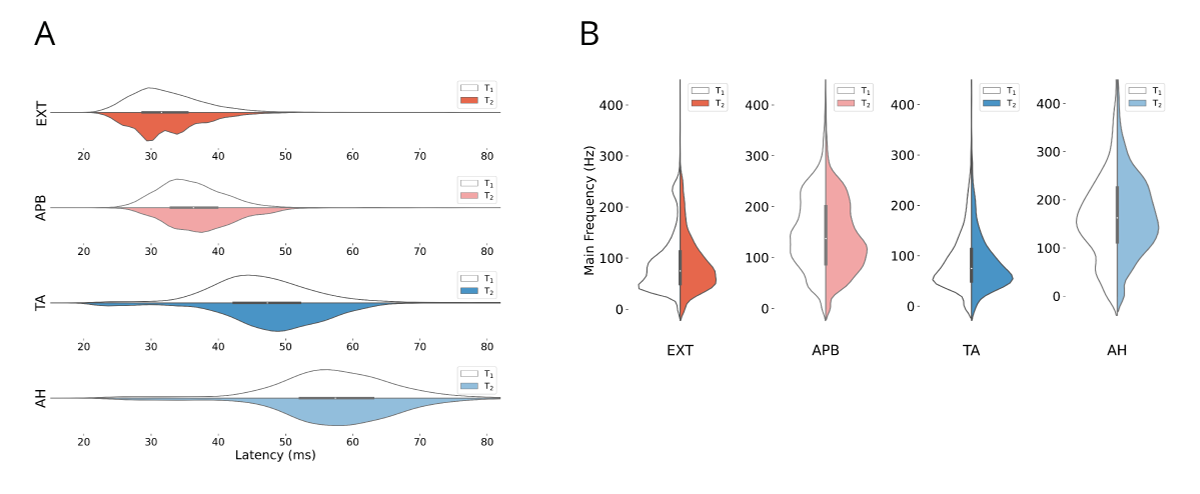
MEP properties across the 2 centers. (A) Latency distribution for both centers and for each muscle. The latencies of all muscles are significantly shorter at center T1. (B) Main frequency distribution for both centers and each muscle. At both centers, the distal muscles (APB and AH) exhibit a higher main frequency than the proximal muscles (EXT and TA). AH: abductor hallucis; APB: abductor pollicis brevis; EXT: extensor digitorum; TA: tibialis anterior.

The distribution of muscle recordings is illustrated in Table 1. A notable discrepancy was observed in the proportion of MEPs recorded from the upper extremities between centers T_1_ (75%, 27,758/36,992) and T_2_ (58.8%, 14,298/24,298). This variation stems from differences in surgical procedures, montage standards, and stimulation techniques. As shown in Table 1, the proportion of MEPs elicited via DCS was significantly higher in center T_1_ (89%, 134/151) than in center T_2_ (30%, 17/58). One striking difference in the MEP data was the significantly shorter onset latencies across all muscles for center T_1_ (*P*<.001; Figure 3B). It is crucial to consider these disparities when interpreting the classification results. Furthermore, Figure 3C reveals differences in main frequency patterns between proximal muscles (EXT and TA) and distal muscles (APB and AH) for both centers, adding another layer of complexity to the MEP data analysis.

With time representation of the MEPs, the RF classifier achieved 87.9% accuracy on the validation dataset from center T_1_ and 80% on the test set from center T_2_. The 1D-CNN achieved a validation accuracy of 87.8% and a test accuracy of 78.4%. On the feature representation, the RF achieved 80.3% validation accuracy and 74.5% test accuracy. Finally, the 2D-CNN achieved 87.2% validation accuracy and 81.9% test accuracy on the time-frequency representation of the MEPs (see Figure 2A). Examination of the confusion matrices (Figure 2C and Multimedia Appendix 2) revealed subtle variations in decision-making patterns across muscles for different data representations and models. Generally, the more high-dimensional data input (time and time-frequency representation) performed better at the classification task than the feature representation of the data. Comparing the performances overall muscles between the RF on the feature representation and the 2D-CNN on the time-frequency representation, the former has a lower overall accuracy than the latter. However, when evaluating performance consistency across muscles, the feature representation demonstrated slightly less variability, evidenced by a lower SD (6.44% for RF on feature representation vs 7.3% for 2D-CNN) and coefficient of variation (8.15 vs 8.56, respectively), of performances.

### Evaluating Decision Confidence

In intraoperative clinical settings, the confidence with which decisions are made is important. To assess this aspect in our classification task, we examined the confidence of our algorithms in categorizing muscles. Figure 2B illustrates the relationship between the proportion of confident predictions (those meeting a specific confidence threshold) and the corresponding test accuracy (see also Multimedia Appendix 2). For a four-class problem, the chance level is 25%. Notably, our models consistently outperform this baseline, showing robust performance. However, as confidence thresholds increase, the proportion of data that meet these criteria diminishes, albeit resulting in enhanced accuracy. The RF models incur a proportionally higher data cost for achieving this accuracy enhancement compared with CNNs.

### Insights Into Model Decision-Making

#### Explicit Feature Representation

**Figure 4:**
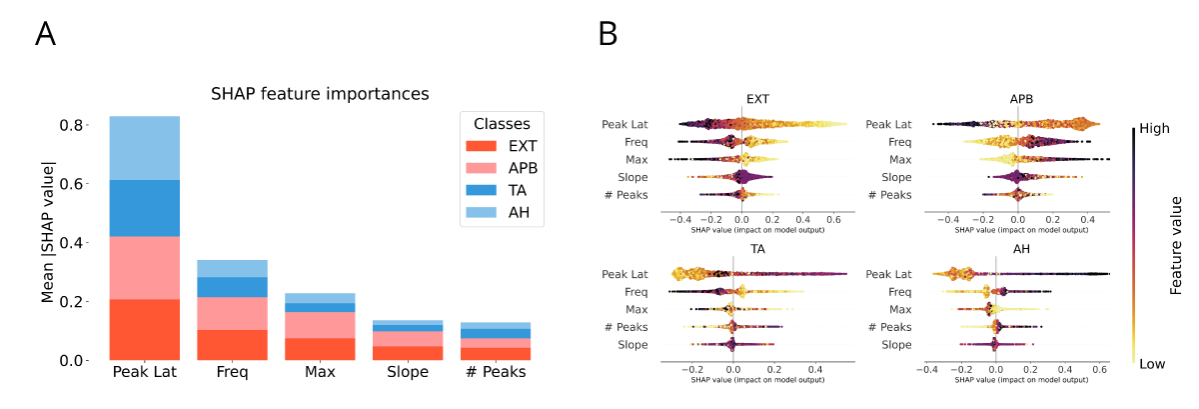
Model decisions according to muscle classification. (A) SHAP feature importances of the RF on feature representation. (B) Beeswarm plot of SHAP values for each muscle classification of the RF on data with feature representation. The features are ordered by importance (top to bottom). Feature values are color-coded (black: high value, yellow: low value). Being on the right (positive SHAP values) means that the feature contributes to the (not necessarily correct) prediction of that particular class. Latency is the most important feature in this situation, with short latencies indicating upper extremity muscles, and long latencies lower extremity muscles. The second most important feature is the main frequency, with high frequencies leading to a decision toward distal muscles (APB and AH), whereas low frequencies push the decision toward proximal muscles (EXT and TA). AH: abductor hallucis; APB: abductor pollicis brevis; EXT: extensor digitorum; SHAP: Shapley Additive Explanation; TA: tibialis anterior.

Although algorithms using explicit feature representations showed poorer performance (80% test accuracy), they provided key insights into the decision-making process. Feature importance analysis (Figure 4A) revealed that peak latency, a primary factor in clinical decision-making, was the main driver of classification. Main frequency was the next most important, despite typically not being used by clinicians. This was followed in order of importance by maximum signal value, while slope and number of peaks were the least important features.

SHAP values from the RF model using feature representation elucidated how different parameters influenced the model’s decisions for each class (Figure 4B). In all four muscles, peak latencies were crucial. For the upper extremity, short latencies favored correct predictions, whereas long latencies were accurate indicators for the lower extremity classes. Interestingly, the main frequencies did not exhibit this pattern. High main frequency values favored predictions for distal muscles (APB and AH), whereas low frequencies were associated with proximal muscles (EXT and TA).

#### Implicit Feature Representation in CNNs

**Figure 5:**
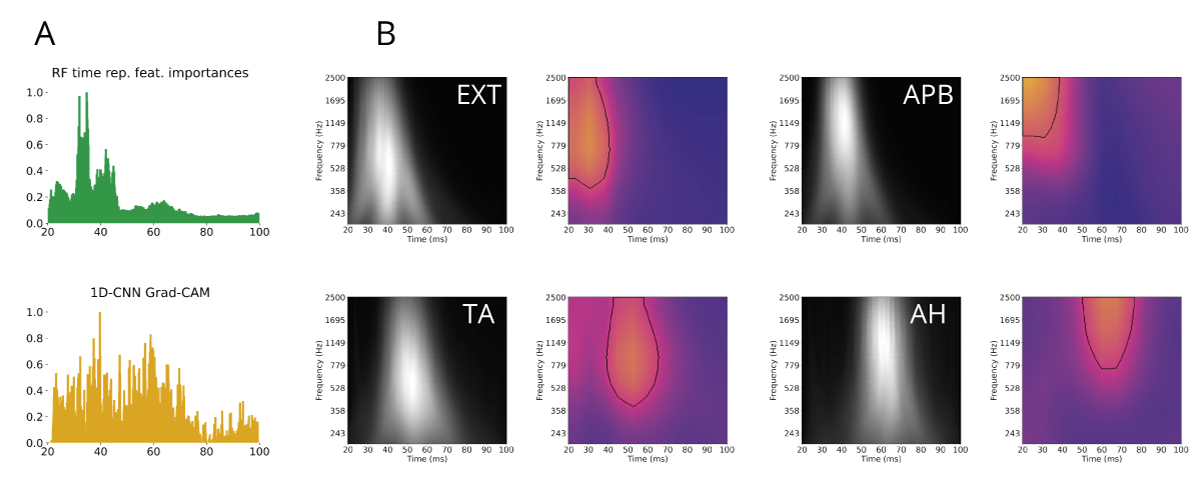
Feature insights for time-series and time-frequency models. (A) Top: The feature importance values of the random forest on time representation motor-evoked potential (MEP) data. Bottom: Averaged Grad-CAM values (over all muscles and all signals) for the 1D convolutional neural network (CNN). In both cases, the most important features are the data points where the MEP occurs (depending on the extremity, between 20 and 60 ms). (B) Average wavelet transform (left, black and white) and average Grad-CAM plots (right, in color) for each muscle. Yellow color and black contour indicate high-attention areas of the CNN. The attention is earlier in the time domain for upper than for lower extremity muscles, whereas the lower bound of the attention contour along the frequency dimension is higher for distal muscles (APB and AH) than for proximal muscles (EXT and TA). AH: abductor hallucis; APB: abductor pollicis brevis; EXT: extensor digitorum; TA: tibialis anterior.

When presented with complex data, such as time-series and wavelet transforms, ML algorithms use internal feature representations to guide their decision-making. For CNNs, these features can be visualized using attention maps, which highlight the areas of the input data that are most salient and decisive for classification. In the depicted Grad-CAMs, these areas correspond to where the signal occurs (see Figure 5B). The insights from explicit feature representation regarding main frequencies are also evident in the Grad-CAMs, since attention for proximal muscles focuses on lower frequencies compared with distal muscles. Similarly, in the feature importance analysis of the RF using the time representation, and in the average (over all signals and all muscles) of the Grad-CAMs of the 1D-CNN, the highest importance is assigned to the time points when the signals occur (see Figure 5A).

## Discussion

### Key Findings

#### Overview

Our study demonstrates the high performance of ML models, such as RF, 1D-CNN, and 2D-CNN, in classifying MEPs recorded during IONM. Notably, the RF classifier achieved 87.9% validation accuracy and 80% test accuracy using time representation data, while the 1D-CNN and 2D-CNN achieved comparable performances with slightly increased variations in accuracy across different datasets.

Furthermore, our analysis revealed that frequency is a critical feature that these algorithms use for decision-making, with different frequency ranges (low vs high frequencies) being decisive depending on the muscle group involved (proximal vs distal, respectively). This is an important finding, which should encourage clinicians to investigate this feature for potential warning criteria. Since there are still disagreements when it comes to warning criteria during intraoperative monitoring of motor evoked potentials, our results may already provide an opportunity to increase patient safety during surgical procedures.

#### Source of Data Differences and Bias in ML Applications

Significant differences in MEP latencies between datasets from centers T_1_ and T_2_ (see Figure 3A) may highlight the influence of different stimulation techniques. The higher number of MEPs induced by DCS at center T_1_ might explain this difference, but other factors might also influence these findings, such as data collection methods, types of surgical procedures, and characteristics of the selected patient population including height, age, disease, etc. For example, the higher proportion of upper extremity MEPs in the data from center T_1_ is due to different surgical focuses at center T_1_ compared with center T_2_. Understanding these differences is crucial for interpreting ML model performance and ensuring generalizability across centers. Including more centers and more extensive data collection can mitigate biases and advance research in the field.

#### Frequency Differences in Proximal Versus Distal Muscle Groups

Our findings indicate significant differences in MEP frequencies between distal and proximal muscle groups. Distal muscles exhibit higher MEP frequencies compared with proximal muscles, a trend that was effectively used by various of our tested models in their decision-making processes. The underlying neurophysiological mechanisms contributing to these differences are not fully understood. Although the general pathway of distal and proximal MEPs are similar—upper motor neurons synapsing on lower motor neurons that innervate muscle fibers at the neuromuscular junction—anatomical and physiological differences between distal and proximal muscles may explain the observed frequency variations. The following physiological and anatomical characteristics outline potential factors contributing to these differences.

First, distal muscles, involved in fine motor control, possess a higher density of smaller motor units compared with proximal muscles. The smaller motor units of distal muscles have lower activation thresholds but generate less force than the larger motor units found in proximal muscles [31-33]. Furthermore, distal hand muscles contain a greater proportion of slow motor units, which are more fatigue-resistant [34,35].

Secondly, the temporal dispersion of electrical activity differs between muscle groups. Distal muscles, such as those in the hand and foot, exhibit more synchronous and temporally concentrated MEP responses, whereas proximal muscles display greater temporal dispersion. This increased synchrony in proximal muscle MEPs likely contributes to the higher frequency distribution observed in distal muscle MEPs.

Thirdly, the cortical representation of distal muscles is significantly larger than that of proximal muscles, reflecting the dense corticospinal innervation of these areas. Hand muscles receive among the strongest corticospinal inputs, highlighting their critical role in precise motor control [36-38].

In addition, various motor control pathways interact differently with distal and proximal muscle groups, further influencing the MEP frequency characteristics. These interactions likely involve contributions from both corticospinal and other descending motor pathways, though their exact contributions require further investigation [39].

The functional relevance of high and low-frequency bands within MEPs remains uncertain. While our findings suggest that MEP characteristics are largely determined by muscle-specific neurophysiology, it is essential to consider the potential role of top-down modulation from cortical and subcortical regions. These central mechanisms could influence observed frequency differences and may contribute to the observed MEP variations between distal and proximal muscles.

Given that intraoperative changes in MEPs are considered critical warning signs of upper and lower motor neuron impairment, further investigation is necessary to clarify the relationship between MEP frequency components and both muscle neurophysiology and neuronal modulation mechanisms.

#### Explaining Decisions: How SHAP and Grad-CAM Uncover MEP Feature Importance

ML models, particularly those designed to handle complex data, consistently achieve high performance, but their lack of transparency can hinder interpretability and therefore acceptance in clinical processes. Specifically, we attained 80% accuracy with our five features per signal, compared with 87% accuracy with 1600 data points per signal. To address interpretability, we applied two complementary explainability techniques: SHAP and Grad-CAM, each offering unique insights into model behavior.

SHAP provides a feature attribution approach, assigning precise numerical contributions to each input feature—such as latency or main frequency—to quantify its role in the prediction process. This method is independent of the choice of ML model and excels at identifying the relative importance of features and offers consistent, interpretable insights, albeit being computationally expensive. In contrast, Grad-CAM generates attention maps that visually highlight which regions of the input signal influenced the model’s decisions. They are model-specific and generally limited to CNNs. These visualizations are particularly useful for validating whether the model focuses on relevant, clinically meaningful areas of the MEP signal.

The combination of SHAP and Grad-CAM allowed us to cross-validate findings, ensuring that the observed importance of main frequency was both quantitatively consistent and visually evident. Compared with other interpretability techniques, such as LIME [40]. SHAP provides more robust and consistent explanations by attributing specific contributions of each feature to individual predictions (local interpretability) while also summarizing feature importance across the entire dataset to reveal overall model behavior (global interpretability).

Our complementary approach highlights the importance of main frequency as a decisive MEP feature and demonstrates how XAI methods can uncover meaningful insights that warrant further testing in basic research and clinical trials. Moreover, the trade-off between explainability and complexity must be carefully considered. In intraoperative settings, where clinical trust is crucial, Grad-CAM’s intuitive attention maps may be favored for transparency. Conversely, SHAP’s precise attributions offer deeper insights for research applications requiring feature-level understanding.

Ultimately, the choice of method depends on the context. Highly accurate yet less interpretable models should not be dismissed if extensively validated on diverse populations. In practice, balancing explainability and accuracy through complementary methods ensures optimal utility, whether for guiding clinical decisions or advancing research.

#### Confidence in Intraoperative Decision-Making and Implications for Clinical Practice

Accurately identifying muscles and avoiding labeling mistakes is critical in IONM, with previous studies highlighting the consequences of such errors that harm the patients we seek to protect [41,42]. During the IONM setup, mislabeling of muscles has caused false negative alarms, in missing MEP alterations of a presumed unaffected muscle. These incidences have caused potentially avoidable motor deficits during surgery and consequently resulted in legal actions. Nevertheless, we have to acknowledge that the IONM setup in the operating room environment is prone to errors as it is a high-pressure environment [43]. Safety checklists have been implemented; however, an automated ML safety check would increase the avoidance of mislabeling. Those algorithms may be implemented in an existing IONM software.

Further, our results suggest that expanding the search for warning criteria to the frequency domain is essential, as different muscles may require tailored approaches. Once these muscle classification models have been validated on more data and more centers, they could be implemented as a safety mechanism at baseline recordings in surgeries.

When analyzing the confidence of our algorithms (see Figure 2B), it became apparent that signal quality and recording modes might limit high-confidence decisions, even with optimal algorithms. This trade-off between data volume and accuracy necessitates either better-trained models or higher-quality recordings. Investing in improved surveillance methods or stable recording techniques, such as averaging or selecting the best MEPs from multiple recordings, could be an essential step. This might affect how MEP monitoring will be done in the future.

Ensuring the trustworthiness of AI involves addressing ethical and legal implications and incorporating decision confidence metrics could bolster acceptance of AI. The question of responsibility is important in a clinical setting and a transparent decision process for any potential implementation of AI is crucial in this regard. This becomes even more evident when discussing legal aspects and accountability. Integrating ML models with robust explainability attributes has the potential to enhance decision-making accuracy. Disclosing the basis of the algorithmic decisions to the neurophysiologists is key, as it allows them to reason how their understanding differs from the algorithm and oversee the intraoperative decision. In our particular MEP muscle identity scenario, it can provide a safety mechanism against muscle mislabeling and facilitate reliable clinical decisions. By elucidating the prediction bases, XAI supports understanding and trust in AI decisions, which is crucial for the seamless implementation of ML tools in real-time surgical environments. This could significantly increase acceptance of AI and its utility in clinical contexts.

### Future Directions and Limitations

Despite the promising results, our study is limited by the small number of centers from which the data originated, potentially introducing center-specific biases. Future research should focus on expanding the dataset to involve more diverse clinical settings and patient populations, thereby improving model robustness and generalizability. This should include different IONM devices, stimulation paradigms, and surgical practices. In addition, further exploration of model interpretability techniques could enhance our understanding of ML decision-making, driving advancements in IONM practices. The next steps would be optimizing feature engineering and investigating changes in MEP features, especially the frequency domain during the surgery to predict motor deficits. As we know from previous studies, these frequency changes occur permanently in patients with deficits [44].

Future research should adopt a structured, multitiered approach to address remaining challenges and to advance the integration of ML-based IONM solutions. Immediate next steps involve expanding datasets, improving feature engineering, and validating models across diverse populations and various centers to enhance robustness and generalizability. Intermediate goals include developing standardized platforms for real-time integration, improving signal quality, and refining XAI frameworks. The long-term vision aims for real-time AI-assisted IONM systems to enhance decision-making, address legal and ethical considerations, and improve surgical safety through large-scale clinical trials and dynamic feedback mechanisms.

### Conclusion

Our study highlights the potential of ML models, including RFs and CNNs, for accurately classifying motor evoked potentials across muscle groups during intraoperative neurophysiological monitoring. By demonstrating robust performance across independent datasets, we underline the reliability and generalizability of these models when applied to complex surgical environments. Importantly, our results identify frequency as a decisive feature, particularly in distinguishing between distal and proximal muscles. While this provides already an intraoperative safety mechanism against mislabeling, our findings have wider implications. This overlooked parameter offers a promising avenue for improving warning criteria during surgeries and providing the opportunity for timely intervention.

Integrating XAI techniques, specifically SHAP and Grad-CAM, provided critical transparency into model decisions. XAI elucidates the underlying prediction bases, enhancing interpretability and fostering clinical trust—key prerequisites for successful deployment in real-time surgical settings. In the context of IONM, this transparency serves as a safety mechanism against muscle mislabeling, a persistent issue that can lead to avoidable motor deficits and legal consequences.

By bridging the gap between model performance, clinical interpretability, and real-world implementation, this research paves the way for broader and more reliable AI applications in IONM-guided surgery. Real-time AI-assisted MEP monitoring holds the potential to transform intraoperative practices by improving decision accuracy, mitigating human error, and safeguarding patient outcomes.

## Data Availability

The datasets analyzed during this study are not publicly available due to anonymity concerns. The models and preprocessing steps used during this study are included in this published article in Multimedia Appendix 4. An intermediate level of expertise is necessary to use the custom-written Python scripts effectively.

## Appendix

Multimedia Appendix 1 Model training.

Multimedia Appendix 2 Additional figures.

Multimedia Appendix 3 TRIPOD-AI (Transparent Reporting of a Multivariable Prediction Model for Individual Prognosis or Diagnosis–Artificial Intelligence) checklist.

Multimedia Appendix 4 Code for model evaluation.

## Abbreviations

AH: abductor hallucis
AI: artificial intelligence
APB: abductor pollicis brevis
CNN: convolutional neural network
DCS: direct cortical stimulation
EXT: extensor digitorum
Grad-CAM: gradient class activation map
IONM: intraoperative neurophysiological monitoring
MEP: motor-evoked potential
ML: machine learning
RF: random forest
SHAP: Shapley Additive Explanation
TA: tibialis anterior
TES: transcranial electrical stimulation
TRIPOD-AI: Transparent Reporting of a Multivariable Prediction Model for Individual Prognosis or Diagnosis–Artificial Intelligence
XAI: explainable artificial intelligence
