# Supplementary material for: Explainable AI for Intraoperative Motor-Evoked Potential Muscle Classification in Neurosurgery: Bicentric Retrospective Study
Source: J Med Internet Res. 2025 Mar 24;27:e63937. doi: 10.2196/63937 (PMC11976170; doi:10.2196/63937)
Supplement: Multimedia Appendix 1 [file jmir_v27i1e63937_app1.docx]

**Supplementary Document 1**

Random forest hyperparameter tuning

The following hyperparameters were used for the grid search of the random forest on time representation of the MEP data:

n_estimators = [200,500,1000]

min_samples_split = [2,16,64]

max_samples = [0.6,0.8]

random_state = [42]

The best parameters, used for the training of the random forests, are:

n_estimators = 1000

min_samples_split = 2

max_samples = 0.8

random_state = 42

1D-CNN architecture

Model: "sequential"

_________________________________________________________________

Layer (type) Output Shape Param #

=================================================================

conv1d (Conv1D) (None, 1594, 8) 64

conv1d_1 (Conv1D) (None, 1588, 8) 456

max_pooling1d (None, 794, 8) 0

(MaxPooling1D)

dropout (Dropout) (None, 794, 8) 0

batch_normalization (None, 794, 8) 32

(BatchNormalization)

flatten (Flatten) (None, 6352) 0

dense (Dense) (None, 512) 3252736

dropout_1 (Dropout) (None, 512) 0

dense_1 (Dense) (None, 4) 2052

=================================================================

Total params: 3,255,340

Trainable params: 3,255,324

Non-trainable params: 16

_________________________________________________________________

2D-CNN architecture

Model: "sequential"

_________________________________________________________________

Layer (type) Output Shape Param #

=================================================================

conv2d (Conv2D) (None, 218, 218, 16) 800

batch_normalization (None, 218, 218, 16) 64

(BatchNormalization)

conv2d_1 (Conv2D) (None, 216, 216, 32) 4640

batch_normalization_1 (None, 216, 216, 32) 128

(BatchNormalization)

max_pooling2d (None, 43, 43, 32) 0

(MaxPooling2D)

conv2d_2 (Conv2D) (None, 41, 41, 64) 18496

batch_normalization_2 (None, 41, 41, 64) 256

(BatchNormalization)

max_pooling2d_1 (None, 13, 13, 64) 0

(MaxPooling2D)

conv2d_3 (Conv2D) (None, 11, 11, 128) 73856

batch_normalization_3 (None, 11, 11, 128) 512

(BatchNormalization)

global_max_pooling2d (None, 128) 0

(GlobalMaxPooling2D)

dense (Dense) (None, 4) 516

=================================================================

Total params: 99268 (387.77 KB)

Trainable params: 98788 (385.89 KB)

Non-trainable params: 480 (1.88 KB)

_________________________________________________________________
