# Supplementary material for: Explainable AI for Intraoperative Motor-Evoked Potential Muscle Classification in Neurosurgery: Bicentric Retrospective Study
Source: J Med Internet Res. 2025 Mar 24;27:e63937. doi: 10.2196/63937 (PMC11976170; doi:10.2196/63937)
Supplement: Multimedia Appendix 2 [file jmir_v27i1e63937_app2.docx]

**Supplementary Figures**


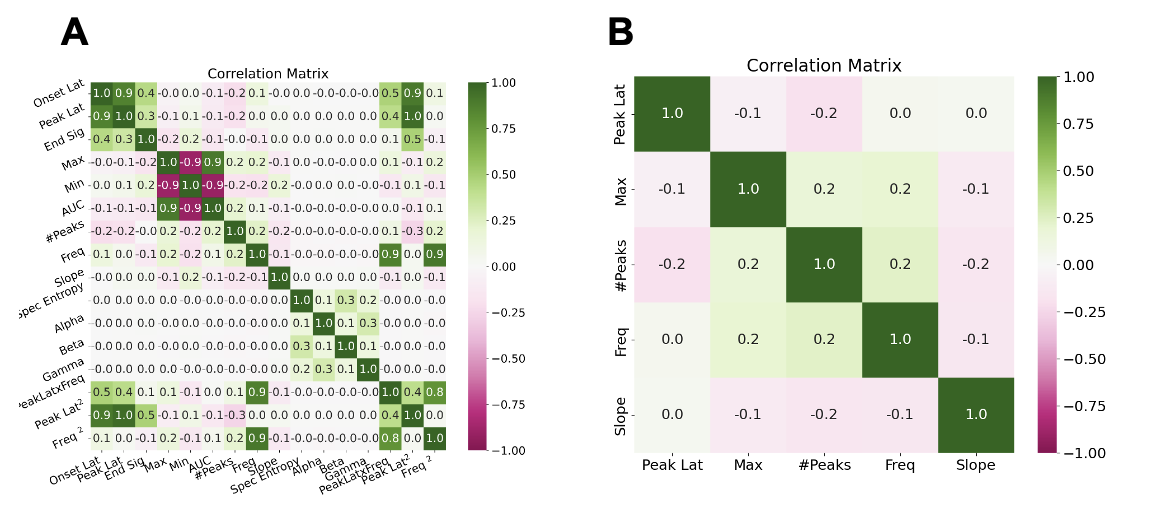


Supplementary Figure 1. Feature exploration. A: Correlation matrix with expansive feature selection of generally used parameters for neurophysiological signal analysis. B: Final subset of selected features.

**
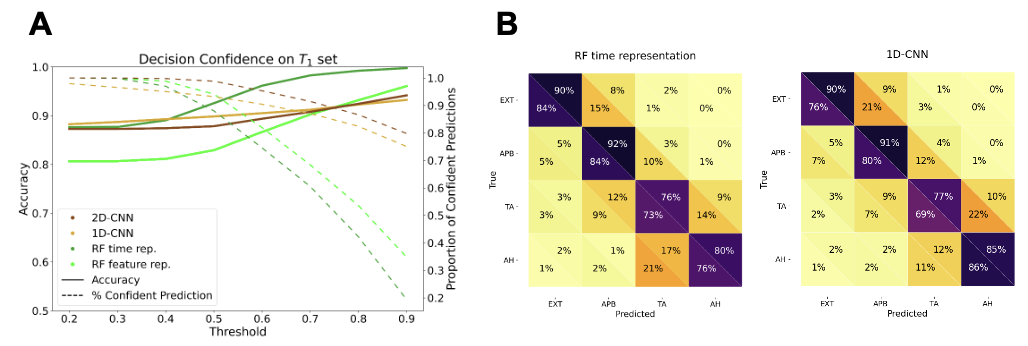
**

Supplementary Figure 2: A: Decision confidence for the training center T1. Solid lines are the accuracies (left y-axis) of the various models for different confidence thresholds. The dashed lines show the proportion of data with these confidences (right y-axis). B: Bicentric confusion matrices: lower triangle (center T2), upper triangle (center T1) for both RF on time representation and 1D-CNN.
